# GLP-1 Receptor Agonists and Diabetic Kidney Disease: A Game Charger in the Field?

**DOI:** 10.3390/life14111478

**Published:** 2024-11-14

**Authors:** Georgia Doumani, Panagiotis Theofilis, Vasilis Tsimihodimos, Rigas G. Kalaitzidis

**Affiliations:** 1 General Hospital of Nikaia-Piraeus Agios Panteleimon, Center for Nephrology “G. Papadakis”, 18454 Piraeus, Greece; geo.doumani@gmail.com (G.D.); panos.theofilis@hotmail.com (P.T.); 2 Department of Internal Medicine, Faculty of Medicine, University of Ioannina, 45110 Ioannina, Greece; vtsimi@uoi.gr

**Keywords:** GLP-1RA, type 2 diabetes mellitus, chronic kidney disease

## Abstract

Kidney disease is a public health epidemic affecting 10% of the population worldwide with a constantly rising incidence, and it is an important contributor to morbidity and mortality. Type 2 diabetes mellitus (T2DM) is a chronic complex condition with a rising incidence worldwide. T2DM remains the principal cause of chronic kidney disease (CKD), which is related to a high risk for cardiovascular (CV) events, end-stage kidney disease (ESKD), and, overall, considerable morbidity and mortality. In the past few decades, various therapeutic treatments have targeted the culprit pathways for slowing CKD progression, with partial success. Thus, despite new advances in patients’ treatment, progressive loss of kidney function or death from T2DM and CKD complications compel new therapeutic pathways. Renin–angiotensin–aldosterone-system-blocking agents have been the only treatment until recently. On top of this, sodium–glucose co-transporter 2 inhibitors along with finerenone showed an impressive ability to reduce the progression of kidney disease and cardiovascular events in diabetic patients with CKD. Glucagon-like peptide-1 receptor agonists (GLP-1RAs) can play a special role and could be a game changer in this field. The latest FLOW trial confirmed multiple favorable clinical effects on renal, cardiovascular, and survival outcomes among high-risk patients treated with semaglutide and supports a significant therapeutic role for GLP-1RAs in this population, although larger-scale evaluation of their risks is needed, given their increasing use.

## 1. Introduction

Kidney disease is a public health epidemic, a risk factor for cardiovascular disease, and a direct cause of morbidity and mortality. In 2017, 1.2 million people died from chronic kidney disease (CKD) and the number of diagnosed, all-stage CKD cases reached 697.5 million [1]. At the same time, the global prevalence of the disease was 9.1% [1]. On the other hand, type 2 diabetes mellitus (T2DM) is a chronic complex condition with a continuously rising incidence worldwide. T2DM remains the leading cause of CKD, which is associated with a high risk for cardiovascular (CV) events, end-stage kidney disease (ESKD), and, overall, considerable morbidity and mortality [2]. Previous reports suggested that the prevalence of CKD in patients with T2DM ranges from 27.1% to 83.6%, while in the US National Health and Nutrition Examination Survey (NHANES) from 2009 to 2014, CKD prevalence was 26.2% [3]. The coexistence of T2DM and renal disease is associated with an additional risk of increased mortality [4].

We know that diabetic kidney disease (DKD) is the end result of multiple pathophysiologic mechanisms, such as glomerular hyperfiltration, inflammation, and fibrosis, resulting in structural and functional alterations in the kidneys of patients with T2DM [5]. Thus, DKD as an entity deserves greater attention. The criteria for the diagnosis and risk stratification of CKD include the estimated glomerular filtration rate (eGFR) as well as the urinary albumin to creatinine ratio (UACR) [6].

We now consider that, at an early stage, CKD progression can be retarded with multiple aggressive therapies [6,7]. Clinical practice guidelines recommend multitudinous interventions including lifestyle changes, modification of diet, salt restriction, body weight reduction, exercise, and diminished alcohol consumption. Until recently, pharmacological treatment approaches in this direction included renin–angiotensin–aldosterone system (RAAS)-blocking agents, namely angiotensin-converting enzyme inhibitors (ACEi) or angiotensin II receptor blockers (ARB) at the maximum tolerated doses [6]. These agents, due to their renoprotective properties, became the medication approved for CKD prevention or progression and have been tried in previous decades with partial success. Indeed, in patients with diabetes, the best example provided is in the Reduction of Endpoints in NIDDM with the Angiotensin II Antagonist Losartan (RENAAL) and Irbesartan Diabetic Nephropathy Trial (IDNT) studies, where treatment with ARBs (losartan and irbesartan) showed a risk reduction for the primary outcome (doubling of serum creatine, ESKD, or death) by 16% and 20%, respectively [8,9]. All the newer treatments should be administered on top of these agents [6]. New hope for improved treatment of CKD was provided with sodium–glucose co-transporter 2 inhibitors (SGLT2i) along with finerenone, the new nonsteroidal mineralocorticoid receptor antagonist for CKD patients at increased risk for CV events [10,11].

In the past few decades, anti-hyperglycemic agents with reported glucose-independent effects have been widely used in the management of T2DM. Among them, SGLT2i was found to exert significant renoprotective actions and is now considered the mainstay of prevention and treatment of CKD in patients with diabetes. In light of these issues, the Dapagliflozin in Patients with Chronic Kidney Disease (DAPA-CKD) study included 4304 participants with a median follow-up of 2.4 years. The study showed that dapagliflozin achieved a significantly lower incidence (9.2%) of the composite endpoint (decline in eGFR, ESKD, or death from renal or cardiovascular causes) compared to the placebo group (14.5%) (hazard ratio (HR), 0.61; 95% confidence interval (CI), 0.51 to 0.72; *p* < 0.001) [12]. These observations were further supported by the Empagliflozin in Patients with Chronic Kidney Disease (EMPA-KIDNEY) study, including 6609 patients with a median of 2.0 years of follow-up. The study showed that treatment with empagliflozin achieved a greater reduction in the progression of kidney disease or cardiovascular death (13.1%) compared to placebo (16.9%) (HR, 0.72; 95% CI, 0.64 to 0.82; *p* < 0.001) [13].

In the category of nonsteroidal mineralocorticoid receptor antagonists, finerenone seems to play a dominant role, targeting inflammation, oxidative stress, and fibrosis [14]. Finerenone in the Efficacy and Safety of Finerenone in Subjects With Type 2 Diabetes Mellitus and Diabetic Kidney Disease (FIDELIO-DKD) and the Efficacy and Safety of Finerenone in Subjects With Type 2 Diabetes Mellitus and the Clinical Diagnosis of Diabetic Kidney Disease (FIGARO-DKD) study, as well as in their pooled analysis, clearly showed reduced kidney failure and mortality and morbidity from CV causes in patients with CKD and T2D [15,16,17].

Τhe fourth pillar for kidney and heart protection, together with RAAS inhibitors, SGLT2i, and finerenone, seems to be glucagon-like peptide-1 receptor agonists (GLP-1RAs). In large-scale clinical trials, GLP-1RAs reduced the incidence of cardiovascular events in diabetic patients at high cardiovascular risk. In these trials, the subgroups of patients with established CKD at baseline experienced similar reductions in cardiovascular events compared to patients with normal renal function. Consequently, recent guidelines suggest using these agents for preventing cardiovascular events in patients with diabetes and CKD [10].

**Figure 1:**
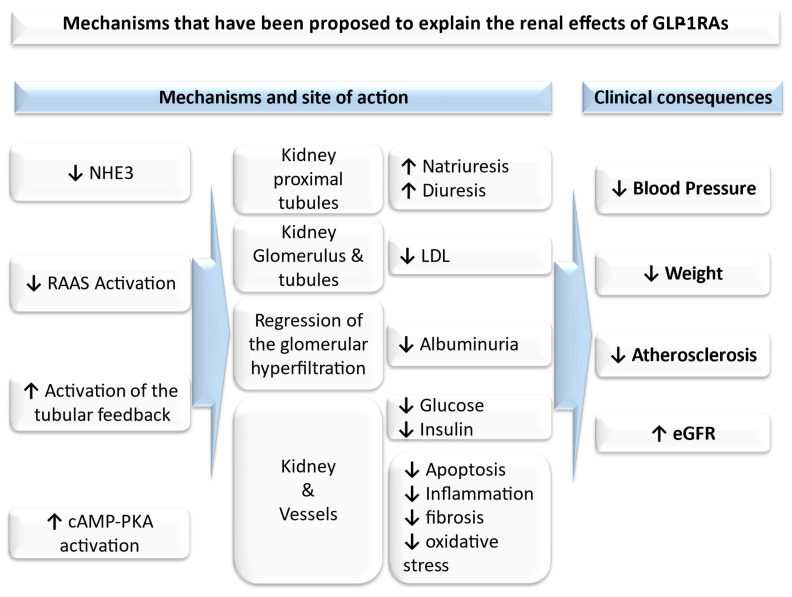
Renal effects of GLP-1RAs (proposed mechanisms). cAMP-PKA: cyclic adenosine monophosphate protein kinase A; eGFR: estimated glomerular filtration rate; LDL: low-density lipoprotein; NHE3: sodium–hydrogen exchanger 3; RAAS: renin–angiotensin–aldosterone system; ↓: reduced; ↑ increased.

Based on the accumulated evidence, recent data suggest that GLP-1RAs may offer renoprotective effects through direct or indirect actions. As several recent studies have shown, GLP-1RAs may prevent the onset of albuminuria and slow the decline in eGFR in patients with T2DM. However, this is only the tip of the iceberg considering their favorable impact (Figure 1) [18,19].

## 2. Literature Search and Selection Criteria

Literature was sourced primarily from PubMed, Embase, and Google Scholar databases, focusing on publications from the past ten years to ensure the inclusion of recent advancements. Keywords used in the search included combinations of “GLP-1 receptor agonists”, “diabetic kidney disease”, “type 2 diabetes”, “renal outcomes”, “cardiovascular outcomes”, “albuminuria”, and “eGFR”. Additionally, landmark studies and pivotal trials were included regardless of publication date to provide foundational insights.

Articles were selected if they met the following criteria:

- Reported on clinical outcomes related to DKD or cardiovascular risk in type 2 diabetes patients treated with GLP-1RAs.
- Included results from randomized controlled trials, large observational studies, meta-analyses, or comprehensive reviews.
- Focused on both glycemic and non-glycemic effects of GLP-1RAs, specifically emphasizing renal and cardiovascular protection.

Two authors independently reviewed the identified articles based on titles and abstracts, followed by a full-text review to ensure relevance and adherence to inclusion criteria. Discrepancies were resolved through consensus or consultation with a third author.

## 3. Glucagon-like Peptide-1

In 1932, the name “incrétine” was first introduced as a substance contained in duodenal mucosal extracts with the ability to lower glucose levels in dogs, supposedly with the increase in insulin secretion (from intestinal secretion of insulin) [20,21]. Nowadays, it has been proven that the oral administration of glucose results in higher insulin secretion compared to intravenous administration due to the presence of such intestinal hormones [19,22].

Incretins are produced by the small intestine in response to nutrient intake. Several incretins are released in humans. The entero-endocrine K-cells produce the glucose-dependent insulinotropic polypeptide (GIP) and the L-cells in the intestine produce GLP-1. The latter is released by L-cells throughout the intestine, most extensively in the distal ileum and colon. Sodium–glucose co-transporter 1 (SGLT1) stimulates the activation of both K- and L-cells in response to luminal glucose absorption, whilst other nutrients can also stimulate them to release incretins [18].

As a carbohydrate metabolism regulator, incretins increase insulin production in a glucose-dependent mode and inhibit pancreatic α-cells to release glucagon. Additionally, they enhance glucose sensitivity in pancreatic β-cells, stimulate their proliferation, and reduce their apoptosis [18,23,24]. GLP-1R agonism also delays gastric emptying, decelerates digestion of carbohydrates, and attenuates postprandial glucose excursions [25]. Overall, GLP-1 appears to play an important role in the regulation of feeding by increasing satiety signals and abridging appetite, which results in a reduction in food intake and weight loss [18].

In circulating GLP-1, the half-life is very short (less than 2 min), and it is rapidly inactivated, largely by dipeptidyl peptidase 4 (DPP-4), but also by other endopeptidases and aminopeptidases. The glucose-lowering effects of GLP-1 are achieved by activating its receptor GLP-1R. In accordance with these physiological traits, GLP-1RAs and DPP-4 inhibitors have been developed to treat hyperglycemia in patients with T2DM. While GLP-1 is synthesized and secreted by neurons in the hindbrain and L-cells of the intestine, GLP-1R is expressed in various organs, namely the brain, lungs, pancreas, heart, kidney, and gastrointestinal tract [18,26].

## 4. GLP-1RAs and the Kidney in T2DM

Experimental studies have long demonstrated the glucose-independent, renoprotective action of GLP-1RAs in rodent models of DKD [23,27,28]. More recent clinical studies have also confirmed their numerous favorable outcomes on the majority of risk factors for CKD retardation, for instance lowering blood pressure (BP), glucose and insulin levels, and promoting weight loss [18].

There has been evidence that GLP-1R mRNA is expressed in proximal tubules and in preglomerular vascular smooth muscle cells in humans [18,29]. The effect of GLP-1 on natriuresis is likely to be due to the inhibition of the sodium–hydrogen exchanger 3 (NHE3) at the brush border of the proximal tubule, which may explain its action on BP [18,30]. Furthermore, in experimental models, GLP-1R agonism has been shown to decrease indicators of renal RAAS activation, such as angiotensin II, preventing the harmful repercussions of triggering RAAS.

Additionally, GLP-1RAs are thought to improve renal hemodynamic function by repressing glomerular hyperfiltration through the activation of tubuloglomerular feedback. Their natriuretic effect, along with their effect on several mechanisms of diabetic nephropathy, such as hyperglycemia, hypertension, and obesity, among others, may be responsible for their combined antialbuminuric effect [18]. Moreover, GLP-1RAs are related to lower low-density lipoprotein (LDL), total cholesterol, and triglycerides levels, improving dyslipidemia [18,31], modulating inflammation at various sites, including the kidneys and the body vessels [18,32], protecting the kidneys from oxidative injury via the cyclic adenosine monophosphate protein kinase A (cAMP-PKA) activation [18,33], and decelerating atherosclerosis through anti-inflammatory and anti-ischemic actions [18,34,35].

## 5. GLP-1RAs and Cardiovascular Risk in Patients with DKD—Clinical Studies: Effects on Albuminuria and GFR

GLP-1RAs have demonstrated significant CV benefits among patients with T2DM at elevated CV risk, including DKD (Table 1) [19]. In the Liraglutide Effect and Action in Diabetes: Evaluation of Cardiovascular Outcome Results (LEADER) trial, which included 9340 patients and compared liraglutide to placebo, a 26% reduction in the de novo onset of albuminuria was observed, as well as a 19% reduction in UACR with liraglutide treatment, along with indistinguishable “hard” renal outcomes between the two groups. Notably, patients with an eGFR <60 mL/min/1.73 m^2^ appeared to have a significantly greater CV benefit from treatment with liraglutide (HR, 0.69; 95% CI, 0.57 to 0.85) than those with an eGFR >60 mL/min/1.73 m^2^ (HR, 0.94; 95% CI, 0.83 to 1.07). In patients with CKD, high event rates were noticed (almost twice as high as those with normal renal function), contributing to this result [18,36].

The Efficacy and Safety of Liraglutide Versus Placebo as Add-on to Glucose-Lowering Therapy in Patients with T2DM and Moderate Renal Impairment (LIRA RENAL) trial investigated the effects of liraglutide on patients with moderate renal impairment and T2DM. In addition to improving glycemic control without a higher risk of hypoglycemia, liraglutide did not affect renal function after 26 weeks [18,37].

A series of 3297 T2DM patients and CV disease or risk factors for CV disease were randomized to semaglutide or placebo (dose of 0.5 or 1 mg once weekly) in the Trial to Evaluate Cardiovascular and Other Long-Term Outcomes with Semaglutide in Subjects with Type 2 Diabetes (SUSTAIN-6). A significant difference was observed between the placebo and semaglutide groups regarding the glycemic control (−0.7% vs. −1.0% for HbA1c) and body weight reduction of the participants (−2.9 kg vs. −4.3 kg). Newly developed or worsening nephropathy incidence was lower in patients treated with semaglutide after a median follow-up of two years (HR, 0.64; 95% CI, 0.46 to 0.88; *p* = 0.005). Additionally, new-onset albuminuria was reduced by semaglutide (semaglutide vs. placebo; 2.5% vs. 4.9%, respectively), as in LEADER. ESKD, renal death, or doubling serum creatinine concentrations to an eGFR 45 mL/min/1.73 m^2^ were not affected. However, the event rate was too low (<1%) to explore these outcomes adequately [38].

According to a post hoc analysis of the LEADER trial and the SUSTAIN 6 study (liraglutide and semaglutide, respectively), the annual reduction in eGFR for patients treated with GLP-1RAs was slower than those taking placebo. Nevertheless, the results were more pronounced in CKD patients with baseline eGFR less than 60 mL/min/1.73 m^2^ [39]. Another analysis from these two trials disclosed an increased likelihood of a 30% decrease in UACR with liraglutide and semaglutide treatment compared with placebo, irrespective of baseline UACR [40].

A total of 6068 patients with T2DM and acute coronary syndrome were randomized to be treated with lixisenatide or placebo in the Evaluation of Cardiovascular Outcomes in Patients with Type 2 Diabetes After Acute Coronary Syndrome (ELIXA) trial. Compared to SUSTAIN-6 and LEADER, lixisenatide resulted in a more modest improvement in glycemic control (−0.27% HbA1c) and body weight (−0.7 kg). Even though lixisenatide outperformed placebo in ELIXA in terms of change in UACR from baseline to 108 weeks (24% vs. 34%, *p* = 0.004), the lixisenatide-driven renal benefit was still attenuated by modest differences in HbA1c levels (0.3%) during the first 3 months (*p* = 0.07), suggesting that glucose may play a role [18,41].

Meanwhile, an exploratory analysis of ELIXA conducted recently examined the effect of lixisenatide on kidney outcomes [42]. Adjusted for baseline HbA1c (HR, 0.808; 95% CI, 0.660 to 0.991; *p* = 0.0404) and on-trial HbA1c (HR, 0.815; 95% CI, 0.665 to 0.999; *p* = 0.0491), lixisenatide-treated patients exhibited a reduced risk of new-onset albuminuria. Among the albuminuric group, the decline in eGFR from baseline was greatest but no significant difference was observed between the two groups at week 108. In addition, no substantial differences in eGFR decline were found between the treatment groups, regardless of the UACR subgroup. Based on the results of the ELIXA trial, renal adverse events were low in both groups (48 (1.6%) of 3032 placebo-treated patients, 48 (1.6%) of 3031 lixisenatide-treated patients). A significant reduction in UACR was observed with lixisenatide after adjustment for HbA1c and other traditional risk factors (metabolic and hemodynamic). According to this exploratory analysis, short-acting GLP-1RAs compared to long-acting GLP-1RAs may exhibit similar effects in the kidney that have been reported previously in CV outcome trials. It is important to note, however, that the higher reduction in systolic BP observed in the SUSTAIN-6 trial (1.3 mmHg for placebo and 2.4 mmHg for semaglutide, respectively) compared to ELIXA (0.8 mmHg) may have an effect on the reduction in albuminuria observed in the SUSTAIN-6 [18].

In the Exenatide Study of Cardiovascular Event Lowering (EXSCEL) trial, 14,752 patients with T2DM with or without previous CV disease were randomly assigned to exenatide (subcutaneous injections of extended-release exenatide at a dose of 2 mg) or placebo once weekly. The patients were monitored for 3.2 years on average. Trial results confirmed the CV safety of once weekly [43]. The renal outcomes of the EXSCEL trial were investigated in a subsequent adjusted analysis. With exenatide added to T2DM patients, research results showed that the composite outcome (40% eGFR decline, renal replacement therapy, renal death, or the onset of albuminuria) was decreased significantly (HR, 0.85; 95% CI, 0.73 to 0.98; *p* = 0.027) [44].

The Dulaglutide versus Insulin Glargine in Patients with Type 2 Diabetes and Moderate-to-Severe Chronic Kidney Disease (AWARD-7) study compared dulaglutide and insulin glargine for glycemic control in patients with T2DM and CKD (moderate to severe). It was an open-label, multicenter trial that involved 577 patients. For 52 weeks, participants were randomly assigned to receive either 1.5 mg of injectable dulaglutide once weekly, 0.75 mg of injectable dulaglutide once weekly, or insulin glargine daily in combination with insulin lispro. HbA1c at 26 weeks was the primary outcome, with a non-inferiority margin of 0.4%, while secondary outcomes included eGFR and UACR values. With dulaglutide 1.5 mg, eGFR levels were higher (least squares mean (LSM) 34.0 mL/min/1.73 m^2^; standard error (SE) 0.7; *p* = 0.005 vs. insulin glargine). eGFR levels were also higher with dulaglutide 0.75 mg (33.8 mL/min/1.73 m^2^ (0.7); *p* = 0.009 vs. insulin glargine) compared to insulin glargine (31.3 mL/min/1.73 m^2^ (0.7)). Furthermore, in comparison with insulin glargine, dulaglutide 1.5 mg and 0.75 mg did not significantly reduce the UACR after 52 weeks. Incident ESKD was noted in 38 patients: 8 patients (4%) of 192 participants treated with dulaglutide 1.5 mg, 14 patients (7%) of 190 participants treated with dulaglutide 0.75 mg, and 16 patients (8%) of 194 participants taking insulin glargine [45]. According to the results of the AWARD-7 study, dulaglutide once weekly produced significant improvements in glycemic control in CKD patients, with effectiveness similar to insulin glargine daily. Based on secondary endpoint analyses, dulaglutide reduced eGFR decline in patients with T2DM and CKD compared to insulin glargine over 52 weeks. The study was the first to show clear effects of a GLP-1RA on eGFR in patients with T2DM and moderate-to-severe CKD [18,46,47].

In accordance with the aforementioned findings, the results of the latest trial, Evaluate Renal Function with Semaglutide Once Weekly (FLOW)—which assessed the efficacy and safety of subcutaneous semaglutide (1.0 mg once weekly) in patients with T2DM and CKD to prevent renal failure, substantial kidney loss, and death by kidney-related or cardiovascular causes—were remarkable. This dedicated kidney outcomes study found that a 1.0 mg once-weekly dose of semaglutide significantly reduced the risk of major kidney events (24% reduction in the primary outcome). Semaglutide also reduced the risk of major CV events and death from any cause (HR, 0.71; 95% CI, 0.56 to 0.89). Impressively, it slowed the annual loss of kidney function by a mean of 1.16 mL/min/1.73 m^2^. Also, at 104 weeks of treatment, UACR was decreased by 40% in the semaglutide group compared to 12% in the placebo group. A post hoc analysis of the change in eGFR from baseline to week 104 revealed an almost identical difference of 3.30 mL/min/1.73 m^2^ (95% CI, 2.43 to 4.17). A difference of 4.10 kg in body weight (95% CI, 3.65 to 4.56) was observed between the semaglutide and placebo groups at week 104. The glycated hemoglobin was reduced by 0.81% more than in the placebo group (95% CI, 0.72 to 0.90), and systolic BP exhibited 2.23 mmHg greater reduction in the semaglutide group compared to placebo (95% CI, 1.13 to 3.33). However, the mean reduction in diastolic BP was 0.78 mmHg greater in the placebo group compared to the semaglutide group (95% CI, 0.16 to 1.41). These benefits could represent important mediators of semaglutide’s effects on kidney, cardiovascular, and survival outcomes among high-risk CKD patients. Taking into account the reassuring safety findings, the study supports a significant therapeutic role for semaglutide in the examined population [48].

## 6. Limitations

There are some limitations to be mentioned. First, this review relies primarily on clinical trial data, which may not fully represent real-world patient populations with comorbid conditions or varied adherence to treatment. Second, we are limited by the availability of long-term outcomes for GLP-1RAs in DKD, as most trials are relatively short-term. Finally, the lack of a systematic approach to reference selection could introduce selection bias, although we aimed to include the most relevant and rigorous studies.

## 7. Conclusions

Diabetic patients with moderate or severe CKD are at high risk for adverse CV and kidney outcomes and require a compound approach. GLP-1RAs can play a special therapeutic role in this field, delaying the progression of kidney disease and lowering the risk of end-stage kidney failure, as well as both kidney and CV mortality. The latest FLOW study confirmed the beneficial effects of semaglutide on kidney, cardiovascular, and survival outcomes among CKD patients with type 2 diabetes, suggesting the advantages of GLP-1RA administration in this high-risk population.

## Figures and Tables

**Table 1:** Clinical trials assessing GLP-1RAs in DKD progression.

Study	Subjects in the Study, *n*	Follow-Up	Treatment	Outcome	Results
LEADER	9340 patients Increased CV risk	Follow-up: 3.8 years	Liraglutide vs. placebo	Rate of kidney function decline	26% reduction in the de novo onset of albuminuria; 19% reduction in UACR
LIRA RENAL	279 patients with T2DM stage 3 CKD	Follow-up: 26 weeks	Liraglutide in moderate renal impairment	Rate of kidney function decline	Liraglutide did not affect renal function
SUSTAIN-6	3297 patients with T2DM and CVD or with other CV risk factors	Follow-up: 2 years	Semaglutide vs. placebo	Rate of kidney function decline	Less frequent occurrence of new nephropathy or worsening nephropathy (HR, 0.64; 95% CI, 0.46 to 0.88; *p* = 0.005)
ELIXA	6068 patients with T2DM and Acute Coronary Syndrome	Follow-up: 108 weeks	Lixisenatide vs. placebo.	Albuminuria progression	Lixisenatide reduces UACR in albuminuric patients
EXSCEL	14,752 patients 73% with CVD and T2DM	Follow-up: 3.2 years	Extended-release exenatide vs. placebo	eGFR decline by 40%, RRT or new onset of macroalbuminuria	Favored exenatide group (HR, 0.85; 95% CI, 0.73 to 0.98, *p* = 0.027)
AWARD-7	577 patients with CKD and T2DM	Follow-up: 52 weeks	Dulaglutide vs. insulin glargine	eGFR and UACR change from baseline	Dulaglutide reduced decline in eGFR with glycemic control similar to insulin glargine
FLOW	3533 patients with T2DM and CKD	Follow-up: 3.4 years	Semaglutide vs. placebo	Kidney disease (a composite, onset of kidney failure, 50% reduction in eGFR or death from kidney- or CV-related causes)	Lower risk (24%) of a primary-outcome event in the semaglutide group. All secondary outcomes favored semaglutide: annual eGFR slope decreased by 1.16 mL/min/1.73 m^2^ (*p* < 0.001); major CV events were 18% lower (HR, 0.82; 95% CI, 0.68 to 0.98; *p* = 0.029); risk of death from any cause was 20% lower (HR = 0.80; 0.67–0.95, *p* = 0.01)

## References

[1] Bikbov B., Purcell C.A., Levey A.S., Smith M., Abdoli A., Abebe M., Adebayo O.M., Afarideh M., Agarwal S.K., Agudelo-Botero M. (2020). Global, Regional, and National Burden of Chronic Kidney Disease, 1990–2017: A Systematic Analysis for the Global Burden of Disease Study 2017. Lancet.

[2] Davies M.J., Aroda V.R., Collins B.S., Gabbay R.A., Green J., Maruthur N.M., Rosas S.E., Del Prato S., Mathieu C., Mingrone G. (2022). Management of Hyperglycaemia in Type 2 Diabetes, 2022. A Consensus Report by the American Diabetes Association (ADA) and the European Association for the Study of Diabetes (EASD). Diabetologia.

[3] Koye D.N., Magliano D.J., Nelson R.G., Pavkov M.E. (2018). The Global Epidemiology of Diabetes and Kidney Disease. Adv. Chronic. Kidney Dis..

[4] Afkarian M., Sachs M.C., Kestenbaum B., Hirsch I.B., Tuttle K.R., Himmelfarb J., de Boer I.H. (2013). Kidney Disease and Increased Mortality Risk in Type 2 Diabetes. J. Am. Soc. Nephrol..

[5] Alicic R.Z., Rooney M.T., Tuttle K.R. (2017). Diabetic Kidney Disease. Clin. J. Am. Soc. Nephrol..

[6] Stevens P.E., Ahmed S.B., Carrero J.J., Foster B., Francis A., Hall R.K., Herrington W.G., Hill G., Inker L.A., Kazancıoğlu R. (2024). KDIGO 2024 Clinical Practice Guideline for the Evaluation and Management of Chronic Kidney Disease. Kidney Int..

[7] Theofilis P., Vordoni A., Kalaitzidis R.G. (2023). Novel Therapeutic Approaches in the Management of Chronic Kidney Disease: A Narrative Review. Postgrad. Med..

[8] Brenner B.M., Cooper M.E., de Zeeuw D., Keane W.F., Mitch W.E., Parving H.-H., Remuzzi G., Snapinn S.M., Zhang Z., Shahinfar S. (2001). Effects of Losartan on Renal and Cardiovascular Outcomes in Patients with Type 2 Diabetes and Nephropathy. N. Engl. J. Med..

[9] Lewis E.J., Hunsicker L.G., Clarke W.R., Berl T., Pohl M.A., Lewis J.B., Ritz E., Atkins R.C., Rohde R., Raz I. (2001). Renoprotective Effect of the Angiotensin-Receptor Antagonist Irbesartan in Patients with Nephropathy Due to Type 2 Diabetes. N. Engl. J. Med..

[10] ElSayed N.A., Aleppo G., Bannuru R.R., Bruemmer D., Collins B.S., Ekhlaspour L., Hilliard M.E., Johnson E.L., Khunti K., Lingvay I. (2024). 11. Chronic Kidney Disease and Risk Management: *Standards of Care in Diabetes—2024*. Diabetes Care.

[11] Rossing P., Caramori M.L., Chan J.C.N., Heerspink H.J.L., Hurst C., Khunti K., Liew A., Michos E.D., Navaneethan S.D., Olowu W.A. (2022). KDIGO 2022 Clinical Practice Guideline for Diabetes Management in Chronic Kidney Disease. Kidney Int..

[12] Heerspink H.J.L., Stefánsson B.V., Correa-Rotter R., Chertow G.M., Greene T., Hou F.-F., Mann J.F.E., McMurray J.J.V., Lindberg M., Rossing P. (2020). Dapagliflozin in Patients with Chronic Kidney Disease. N. Engl. J. Med..

[13] Herrington W.G., Staplin N., Wanner C., Green J.B., Hauske S.J., Emberson J.R., Preiss D., Judge P., Mayne K.J., The EMPA-KIDNEY Collaborative Group (2023). Empagliflozin in Patients with Chronic Kidney Disease. N. Engl. J. Med..

[14] Chen W., Zheng L., Wang J., Lin Y., Zhou T. (2023). Overview of the Safety, Efficiency, and Potential Mechanisms of Finerenone for Diabetic Kidney Diseases. Front. Endocrinol..

[15] Filippatos G., Anker S.D., Böhm M., Gheorghiade M., Køber L., Krum H., Maggioni A.P., Ponikowski P., Voors A.A., Zannad F. (2016). A Randomized Controlled Study of Finerenone vs. Eplerenone in Patients with Worsening Chronic Heart Failure and Diabetes Mellitus and/or Chronic Kidney Disease. Eur. Heart J..

[16] Pitt B., Filippatos G., Agarwal R., Anker S.D., Bakris G.L., Rossing P., Joseph A., Kolkhof P., Nowack C., Schloemer P. (2021). Cardiovascular Events with Finerenone in Kidney Disease and Type 2 Diabetes. N. Engl. J. Med..

[17] Bakris G.L., Agarwal R., Anker S.D., Pitt B., Ruilope L.M., Nowack C., Kolkhof P., Ferreira A.C., Schloemer P., Filippatos G. (2019). Design and Baseline Characteristics of the Finerenone in Reducing Kidney Failure and Disease Progression in Diabetic Kidney Disease Trial. Am. J. Nephrol..

[18] Greco E., Russo G., Giandalia A., Viazzi F., Pontremoli R., De Cosmo S. (2019). GLP-1 Receptor Agonists and Kidney Protection. Medicina.

[19] Michos E.D., Bakris G.L., Rodbard H.W., Tuttle K.R. (2023). Glucagon-like Peptide-1 Receptor Agonists in Diabetic Kidney Disease: A Review of Their Kidney and Heart Protection. Am. J. Prev. Cardiol..

[20] Thomas M.C., Coughlan M.T., Cooper M.E. (2023). The Postprandial Actions of GLP-1 Receptor Agonists: The Missing Link for Cardiovascular and Kidney Protection in Type 2 Diabetes. Cell Metab..

[21] La Barre J. (1932). Sur Les Possibilities d’un Traitement Du Diabete Par L’incretine. Bull. Acad. R. Med. Belg..

[22] Yu J.H., Park S.Y., Lee D.Y., Kim N.H., Seo J.A. (2022). GLP-1 Receptor Agonists in Diabetic Kidney Disease: Current Evidence and Future Directions. Kidney Res. Clin. Pr..

[23] Thomas M.C. (2017). The Potential and Pitfalls of GLP-1 Receptor Agonists for Renal Protection in Type 2 Diabetes. Diabetes Metab..

[24] Baggio L.L., Drucker D.J. (2007). Biology of Incretins: GLP-1 and GIP. Gastroenterology.

[25] Goldney J., Sargeant J.A., Davies M.J. (2023). Incretins and Microvascular Complications of Diabetes: Neuropathy, Nephropathy, Retinopathy and Microangiopathy. Diabetologia.

[26] Thornberry N.A., Gallwitz B. (2009). Mechanism of Action of Inhibitors of Dipeptidyl-Peptidase-4 (DPP-4). Best Pract. Res. Clin. Endocrinol. Metab..

[27] Hendarto H., Inoguchi T., Maeda Y., Ikeda N., Zheng J., Takei R., Yokomizo H., Hirata E., Sonoda N., Takayanagi R. (2012). GLP-1 Analog Liraglutide Protects against Oxidative Stress and Albuminuria in Streptozotocin-Induced Diabetic Rats via Protein Kinase A-Mediated Inhibition of Renal NAD(P)H Oxidases. Metabolism.

[28] Kodera R., Shikata K., Kataoka H.U., Takatsuka T., Miyamoto S., Sasaki M., Kajitani N., Nishishita S., Sarai K., Hirota D. (2011). Glucagon-like Peptide-1 Receptor Agonist Ameliorates Renal Injury through Its Anti-Inflammatory Action without Lowering Blood Glucose Level in a Rat Model of Type 1 Diabetes. Diabetologia.

[29] Pyke C., Heller R.S., Kirk R.K., Ørskov C., Reedtz-Runge S., Kaastrup P., Hvelplund A., Bardram L., Calatayud D., Knudsen L.B. (2014). GLP-1 Receptor Localization in Monkey and Human Tissue: Novel Distribution Revealed With Extensively Validated Monoclonal Antibody. Endocrinology.

[30] Gutzwiller J.-P., Tschopp S., Bock A., Zehnder C.E., Huber A.R., Kreyenbuehl M., Gutmann H., Drewe J., Henzen C., Goeke B. (2004). Glucagon-Like Peptide 1 Induces Natriuresis in Healthy Subjects and in Insulin-Resistant Obese Men. J. Clin. Endocrinol. Metab..

[31] Farr S., Taher J., Adeli K. (2014). Glucagon-Like Peptide-1 as a Key Regulator of Lipid and Lipoprotein Metabolism in Fasting and Postprandial States. Cardiovasc. Hematol. Disord. -Drug Targets.

[32] Lee Y.-S., Jun H.-S. (2016). Anti-Inflammatory Effects of GLP-1-Based Therapies beyond Glucose Control. Mediat. Inflamm..

[33] Deb D.K., Bao R., Li Y.C. (2017). Critical Role of the CAMP-PKA Pathway in Hyperglycemia-induced Epigenetic Activation of Fibrogenic Program in the Kidney. FASEB J..

[34] Vinué Á., Navarro J., Herrero-Cervera A., García-Cubas M., Andrés-Blasco I., Martínez-Hervás S., Real J.T., Ascaso J.F., González-Navarro H. (2017). The GLP-1 Analogue Lixisenatide Decreases Atherosclerosis in Insulin-Resistant Mice by Modulating Macrophage Phenotype. Diabetologia.

[35] Sourris K.C., Ding Y., Maxwell S.S., Al-sharea A., Kantharidis P., Mohan M., Rosado C.J., Penfold S.A., Haase C., Xu Y. (2024). Glucagon-like Peptide-1 Receptor Signaling Modifies the Extent of Diabetic Kidney Disease through Dampening the Receptor for Advanced Glycation End Products–Induced Inflammation. Kidney Int..

[36] Marso S.P., Daniels G.H., Brown-Frandsen K., Kristensen P., Mann J.F.E., Nauck M.A., Nissen S.E., Pocock S., Poulter N.R., Ravn L.S. (2016). Liraglutide and Cardiovascular Outcomes in Type 2 Diabetes. N. Engl. J. Med..

[37] Davies M.J., Bain S.C., Atkin S.L., Rossing P., Scott D., Shamkhalova M.S., Bosch-Traberg H., Syrén A., Umpierrez G.E. (2016). Efficacy and Safety of Liraglutide Versus Placebo as Add-on to Glucose-Lowering Therapy in Patients With Type 2 Diabetes and Moderate Renal Impairment (LIRA-RENAL): A Randomized Clinical Trial. Diabetes Care.

[38] Marso S.P., Bain S.C., Consoli A., Eliaschewitz F.G., Jódar E., Leiter L.A., Lingvay I., Rosenstock J., Seufert J., Warren M.L. (2016). Semaglutide and Cardiovascular Outcomes in Patients with Type 2 Diabetes. N. Engl. J. Med..

[39] Perkovic V., Bain S., Bakris G., Buse J., Gondolf T., Idorn T., Lausvig N., Mahaffey K., Marso S., Nauck M. (2019). FP482EGFR loss with glucagon-like peptide-1 (GLP-1) analogue treatment: Data from SUSTAIN 6 and LEADER. Nephrol. Dial. Transplant..

[40] Perkovic V., Bain S., Bakris G., Buse J., Idorn T., Mahaffey K., Marso S., Nauck M., Pratley R., Rasmussen S. (2019). FP483EFFECTS of semaglutide and liraglutide on urinary albumin-to-creatinine ratio (UACR)—A pooled analysis of sustain 6 and leader. Nephrol. Dial. Transplant..

[41] Pfeffer M.A., Claggett B., Diaz R., Dickstein K., Gerstein H.C., Køber L.V., Lawson F.C., Ping L., Wei X., Lewis E.F. (2015). Lixisenatide in Patients with Type 2 Diabetes and Acute Coronary Syndrome. N. Engl. J. Med..

[42] Muskiet M.H.A., Wheeler D.C., Heerspink H.J.L. (2019). New Pharmacological Strategies for Protecting Kidney Function in Type 2 Diabetes. Lancet Diabetes Endocrinol..

[43] Holman R.R., Bethel M.A., Mentz R.J., Thompson V.P., Lokhnygina Y., Buse J.B., Chan J.C., Choi J., Gustavson S.M., Iqbal N. (2017). Effects of Once-Weekly Exenatide on Cardiovascular Outcomes in Type 2 Diabetes. N. Engl. J. Med..

[44] Bethel M.A., Mentz R.J., Merrill P., Buse J.B., Chan J.C., Goodman S.G., Iqbal N., Jakuboniene N., Katona B.G., Lokhnygina Y. (2018). Renal Outcomes in the EXenatide Study of Cardiovascular Event Lowering (EXSCEL). Diabetes.

[45] Tuttle K.R., Lakshmanan M.C., Rayner B., Busch R.S., Zimmermann A.G., Woodward D.B., Botros F.T. (2018). Dulaglutide versus Insulin Glargine in Patients with Type 2 Diabetes and Moderate-to-Severe Chronic Kidney Disease (AWARD-7): A Multicentre, Open-Label, Randomised Trial. Lancet Diabetes Endocrinol..

[46] Georgianos P.I., Vaios V., Roumeliotis S., Leivaditis K., Eleftheriadis T., Liakopoulos V. (2022). Evidence for Cardiorenal Protection with SGLT-2 Inhibitors and GLP-1 Receptor Agonists in Patients with Diabetic Kidney Disease. J. Pers. Med..

[47] Vitale M., Haxhi J., Cirrito T., Pugliese G. (2020). Renal Protection with Glucagon-like Peptide-1 Receptor Agonists. Curr. Opin. Pharmacol..

[48] Perkovic V., Tuttle K.R., Rossing P., Mahaffey K.W., Mann J.F.E., Bakris G., Baeres F.M.M., Idorn T., Bosch-Traberg H., Lausvig N.L. (2024). Effects of Semaglutide on Chronic Kidney Disease in Patients with Type 2 Diabetes. N. Engl. J. Med..

